# Metabolomic Approach in Anticancer Biomarker Discovery from Foliose Lichens

**DOI:** 10.34172/apb.43220

**Published:** 2024-12-13

**Authors:** Chintya Permata Zahky Sukrisno Putri, Dinar Mutia Rani, Ludmilla Fitri Untari, Banun Kusumawardani, Anang Kurnia, Paul A. Keller, Ari Satia Nugraha

**Affiliations:** ^1^Drug Utilisation and Discovery Research Group, Faculty of Pharmacy, Universitas Jember, Jember, Indonesia 68121.; ^2^Department of Tropical Biology, Faculty of Biology, Gadjah Mada University, Yogyakarta 55281, Indonesia.; ^3^Department of Biomedical Sciences, Faculty of Dentistry, University of Jember, Jawa Timur 68121, Indonesia.; ^4^Department of Statistics, IPB University, Jl. Meranti Wing 22 Level 4 Kampus IPB Darmaga, Bogor - Indonesia 16680.; ^5^School of Chemistry and Molecular Bioscience, University of Wollongong, Wollongong, New South Wales, 2522, Australia.

**Keywords:** Lichen, Anticancer, Metabolomics, HeLa, P. caroliniana

## Abstract

**Purpose:**

Lichens are well-known as a source of pharmacologically active compounds. This includes anticancer compounds which have biomass constraints including using traditional techniques of lichen bioprospecting. This current study reports the use of cutting-edge metabolomics and a computational approach to discover anticancer biomarkers from Indonesian lichens.

**Methods:**

Seven lichen crude extracts were evaluated against cervical cell lines HeLa using a MTT assay and secondary metabolites were profiled and recorded via a gas chromatography-mass spectrometry (GC-MS) protocol. A multivariate analysis orthogonal partial least-squares-discriminant analysis (OPLS-DA) was employed to determine anticancer biomarker of the lichens. A structure-based computational study against the HeLa cancer cell related protein targets (BCL-2 (4MAN), AKT-1 (4GV1), MCL-1 (5FDO), and BRAF (5VAM)) was used to determine the most potent biomarker.

**Results:**

The MTT assessment indicated the seven lichens possessed strong, medium and weak cytotoxicity. Multivariate analysis showed an OPLS-DA score plot with distinct separation among the strong, medium and weak cytotoxic groups. The biplot OPLS-DA and GC-MS analysis proposed 13 compounds of *Parmelia caroliniana* and 12 compounds of *Physcia cf. millegrana* as anticancer biomarker candidates. Docking experiments revealed 6-amino-3,4,7-triphenylpyrido[2’,3’:4,5]thieno[2,3-*c*]pyridazine **4** from *P. caroliniana* to possess the highest binding affinity against BCL-2 (4MAN), AKT-1 (4GV1), MCL-1 (5FDO), and BRAF (5VAM) proteins with affinity energy values of -10.0, -11.6, -10.4, -12.6, respectively.

**Conclusion:**

The study successfully revealed compound **4** as the anticancer biomarker against HeLa cell cancer of *P. caroliniana* in which can be further explored through *in vitro* and *in vivo* studies. Further, the metabolomic protocol established can be adapted as a tool for biomarker discoveries from other medicinal plants.

## Introduction

 Cervical cancer is a life-threatening disease with a total of 662 301 cases resulting in 348 874 deaths in 2022.^1^ Complications and side effects of currently available therapeutic agents demand new strategies for the development of new treatments, including the discovery of new anticancer agents with less side effects and minimal cost.^2^ Natural products are known as the source for 60% of the currently available anticancer drugs and these include bleomycin sulfate and topotecan hydrochloride, both natural product derivates used in cervical cancer treatment.^3^ A diversity of organisms have been subjected to anti cervical cancer bioprospecting including the cosmopolitan composite organism, the lichens, which have been used traditionally to treat cancer within numerous cultures around the world.^4^ Numerous lichen secondary metabolites have been isolated and evaluated for their bioactivities against an array of malignant cell lines.^5^

 However, lichen bioprospecting remains a challenging task due to its limited biomass availability.^6^ This restraint traditional bioassay guided phytochemical investigation unsuitable. Therefore, a new mean metabolomic approach is necessary as this requires a relatively small amount of sample.^7^ In this study, a multivariate technique, orthogonal partial least-squares-discriminant analysis (OPLS-DA) was adopted to identify bioactive compounds from selected lichen species which are responsible for their cytotoxicity against HeLa cell line. An *in-silico* study was performed to confirm the anticancer biomarkers.

## Material and Method

###  Lichen collection and extraction

 Seven foliose lichen samples were collected from East Java, Indonesia including *Parmelia aurulenta* Tuck. from Jember district, *Parmelia caroliniana* Nyl. from Pasuruan district, *Parmelia cetrata* Ach., *Parmelia dilatata* Vain., *Cladonia scabriuscula*(Duby) Leight, *Candelaria fibrosa* (Fr.) Müll. Arg., and *Physcia cf. millegrana* Degel. from Bondowoso district. Samples were stored and labelled at the Drug Utilisation and Discovery Research Group (DUDRG), Faculty of Pharmacy, University of Jember, Indonesia. Dried lichen samples were ground in the presence of liquid nitrogen and were then extracted with methanol followed by vacuum drying to produce crude methanol extracts.

###  Anticancer bioassay

 Cytotoxicity was measured using a standard MTT assay.^8^ HeLa (ATCC CRM-CCL-2) cells were treated with serial concentrations of lichen extracts (1024, 512, 256, 128, 64, 32 µg/mL).

###  Metabolomics experiment

 Metabolomic profiles were generated based on a standard gas chromatography-mass spectrometry (GC-MS) protocol developed by Lisec at al.^9^ Compound annotation was generated from spectral data comparison against the mass spectra library, NIST version 2.2.

 Metabolomic studies were conducted using the multivariate analysis software SIMCA by MKS UMETRICS. The data used comprised of the x-axis representing the area at a specific retention time with the y-axis defining the lichen species. Chromatograms were pre-processed to produce 58 binned data and the lichens were classified into 3 groups based on cytotoxicity (IC_50_ values). Multivariate analysis was performed using the OPLS-DA method.

###  Computational study

 Biomarker candidates were investigated using AutoDock Vina v1.2.3 on the HeLa related protein targets BCL-2 (PDB ID: 4MAN), MCL-1 (PDB ID: 5FDO), AKT-1 (PDB ID: 4GV1) and BRAF (PDB ID: 5VAM) with respective positive controls, 1Y1, 5X2, 0XZ and 92J. Molecular energy minimisation and format conversion into pdbqt were performed using ChemBio and MGLTools software 1.5.7., respectively. The best docking conformations were imported and their interactions were evaluated using BIOVIA Discovery Visualizer v21.1.0.20298.

## Results and Discussion

 Lichen secondary metabolites were extracted from seven foliose lichen species using methanol with molecule derivatisation conducted using *N*-methyl trifluoroacetamide (MSTFA) to enable robust molecular detection in the GC-MS. The cytotoxicity bioassay indicated HeLa cell lines to possess various sensitivities against the seven-lichens with *P. millegrana* showing the highest activity (Table 1).

**Table 1:** Cytotoxicity of seven lichens crude extracts against HeLa cell line

**Samples**	**IC**_50_ ** (μg/mL)**	**Group**
*P. millegrana*	137	1
*P. caroliniana*	328	1
*C. scabriuscula*	476	2
*P. aurulenta*	552	2
*C. fibrosa*	733	3
*P. cetrata*	751	3
*P. dilatata*	981	3

Note: Green, blue and red indicate strong, medium and weak cytotoxicity, respectively.

**Figure 1:**
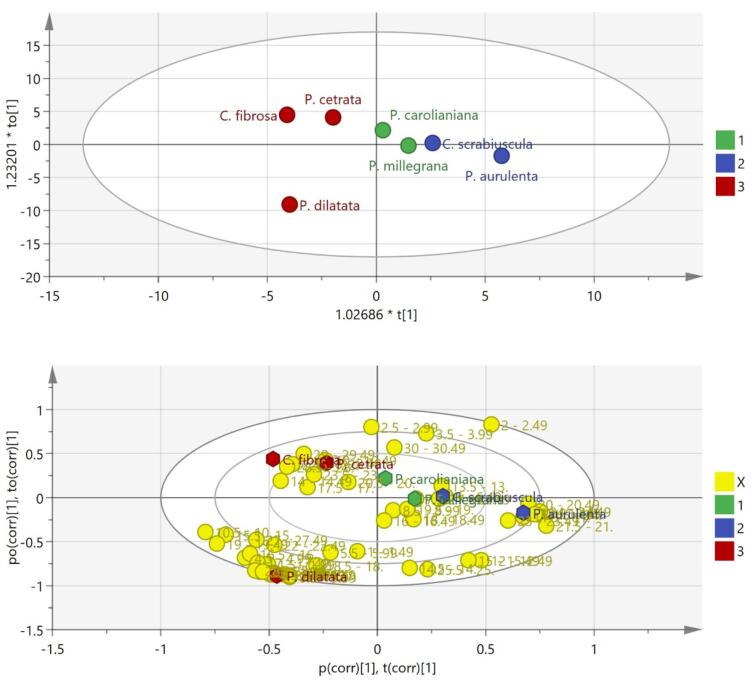
OPLS-DA score plot (top), Biplot OPLS-DA indicating significant variables (retention time binning) of *P. caroliniana* and *P. millegrana*

**Figure 2:**
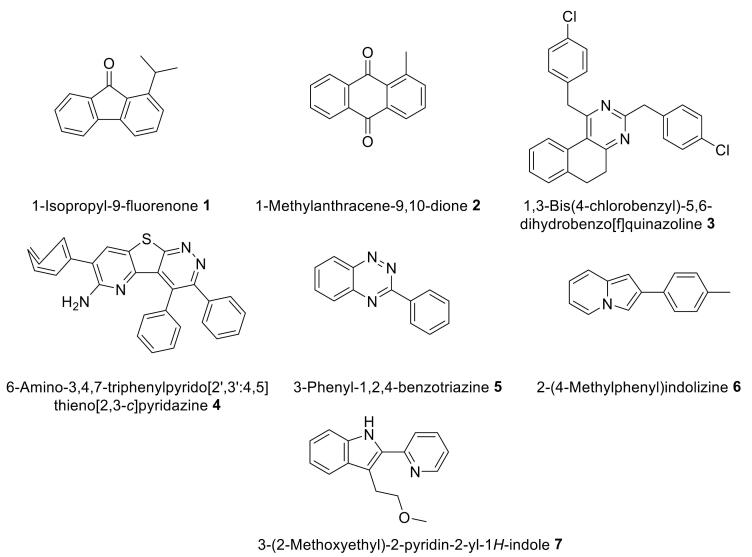
Constituents of *P. caroliniana* with significant affinity against HeLa cell line apoptotic related protein

 A multivariate analysis using OPLS-DA produced good separation between the classified groups as depicted in both 2D score plots (Figure 1). The least active groups (red) including *C. fibrosa*, *P. cetrata* and *P. dilatata* were all gathered in the left quadrant with the medium (blue dots) and active groups (green dots) in the right quadrant (Figure 2, top). Further analysis based on a biplot diagram, an overlayed score and loading plot, displayed a distinct distribution of loading plot (yellow dots, represents the retention time generated from GC-MS chromatogram) around *P. caroliniana* and *P. millegrana* plots (Figure 2, bottom). This biplot analysis facilitated in determining important variables (retention time) which contribute to the anticancer activity of *P. caroliniana* (13.5-13.99; 20.5-20,99; 30-30.49 min) and *P. millegrana* (7.5-7.99; 8.5-8.99; 11.5-11.99; 13.5-13.99; 16-16.49; 18-18.49; 19.5 min). GC-MS compound annotation led to molecular identification of 13 secondary metabolites of *P. caroliniana*and 12 secondary metabolites of *P. millegrana*which correlated to cytotoxicity.

 The *in-silico* investigation on 25 compounds of *P. millegrana* and *P. caroliniana* generated from OPLSDA multivariate analysis was performed using a docking approach with proteins related to cervical cancer, B-cell lymphoma-2 protein (BCL-2, PDB ID: 4MAN), alpha kinase threonin-1 protein (AKT-1, PDB ID: 4GV1), myeloid cell leukemia-1 protein (MCL-1, PDB ID: 5FDO), and V-raf murine sarcoma viral oncogene homolog B1 protein (BRAF, PDB ID: 5VAM) (Table 2). The docking experiment revealed 12 metabolites of *P. millegrana*to possess insignificant binding energy towards all four proteins compared to the binding affinity of corresponding endogenous ligands. In contrast, compounds **1-7** of *P. caroliniana* showed binding affinities comparative to the endogenous ligands that bind 4GV1, 5FDO and 5VAM proteins. None of the secondary metabolites produced significant interactions against the BCL-2 (4MAN) protein.

**Table 2:** Affinity energy of selected secondary metabolites against anti-apoptotic protein BCL-2 (4MAN), AKT-1 (4GV1), MCL-1 (5FDO), and BRAF (5VAM)

**No.**	**Compound**	**Affinity (kcal/mol)**			
		**BCL-2 (4MAN)**	**AKT-1 (4GV1)**	**MCL-1 (5FDO)**	**BRAF (5VAM)**
***P. *** ***caroliniana***					
1	5-methyl-1*H*-[1,2,4]triazolo[1,5-*a*]pyrimidin-7-one	-5.5	-6.5	-5.8	-6.6
2	ethyl 5-(1*H*-benzimidazol-2-ylsulfanylmethyl)-3-ethyl-2-oxooxolane-3-carboxylate	-6.7	-8.2	-6.3	-7.5
3	1-isopropyl-9-fluorenone **1**	-8	-8.6	-8.2	-9.4
4	1-methylanthracene-9,10-dione **2**	-8.2	-9.1	-8.6	-9.6
5	3-methoxy-2,4-dimethoxycarbonyl-5-(methoxypropyl)phenol	-5.7	-6.2	-6	-6.8
6	1,3-bis(4-chlorobenzyl)-5,6-dihydrobenzo[f]quinazoline **3**	-9.4	-8.8	-8.9	-10
7	6-amino-3,4,7-triphenylpyrido[2',3':4,5]thieno[2,3-*c*]pyridazine **4**	-10	-11.6	-10.4	-12.6
8	6-[*N*-(amino)imino]aminomethyl-2,3,5-trichloro-1,4-benzoquinone	-5.9	-7	-6.2	-6.6
9	2-(4-hydroxy-3-methoxyphenyl)acetic acid	-5.6	-6.6	-5.8	-6.2
10	1,2-benzothiazol-3-amine	-5.9	-6.1	-5.8	-6.1
11	3-phenyl-1,2,4-benzotriazine **5**	-7.8	-7.5	-8.8	-8.8
12	2-(4-methylphenyl)indolizine **6**	-7.6	-7.5	-9.2	-8.6
13	3-(2-methoxyethyl)-2-pyridin-2-yl-1*H*-indole **7**	-7.2	-7.7	-7.6	-8.2
***P. *** ***millegrana***					
1	propane-1,2,3-triol	-3.4	-3.9	-3.1	-4.5
2	butane-1,2,3-triol	-3.7	-4.5	-3.6	-5.1
3	(2*R*,3*R*)-2,3,4-trihydroxybutanal	-4	-4.7	-3.6	-4.9
4	butane-1,2,3,4-tetraol	-3.9	-4.8	-3.6	-5.5
5	(*E*)-2,3,4,5-tetrahydroxypentanal *O*-methyl oxime	-5	-5	-	-5.1
6	(*Z*)-1,3,4,5,6-pentahydroxyhexan-2-one *O*-methyl oxime	-4.3	-5.3	-4.4	-5
7	(*Z*)-2,3,4,5,6-pentahydroxyhexanal *O*-methyl oxime	-4.8	-5.7	-4.7	-4.9
8	4-(2-chloroethyl)-1-(2,4-dinitrophenyl)-3,5-dimethyl-1*H*-pyrazole	-6.8	-7.2	-7	-7.2
9	cyclohexane-1,2,3,4,5,6-hexol	-4.6	-5.1	-4.4	-5.6
10	(3*R*,4*R*,5*R*)-oxane-2,3,4,5-tetrol	-4.4	-5.7	-4.5	-5
11	(2*R*,3*S*,4*R*,5*S*)-hexane-1,2,3,4,5,6-hexol	-4.2	-5.5	-4.2	-5.8
12	(*E*)-1,4,5,6-tetrahydroxy-3-((3,4,5-trihydroxy-6-(hydroxymethyl)tetrahydro-2*H*-pyran-2-yl)oxy)hexan-2-one *O*-methyl oxime	-5.6	-6.6	-4.8	-5.2

Note: Affinity of endogenous ligands of the four proteins (4MAN, 4GV1, 5FDO, 5VAM), were -11.1, -8.8, -7.3, and -10.1 kcal/mol, respectively.

**Figure 3:**
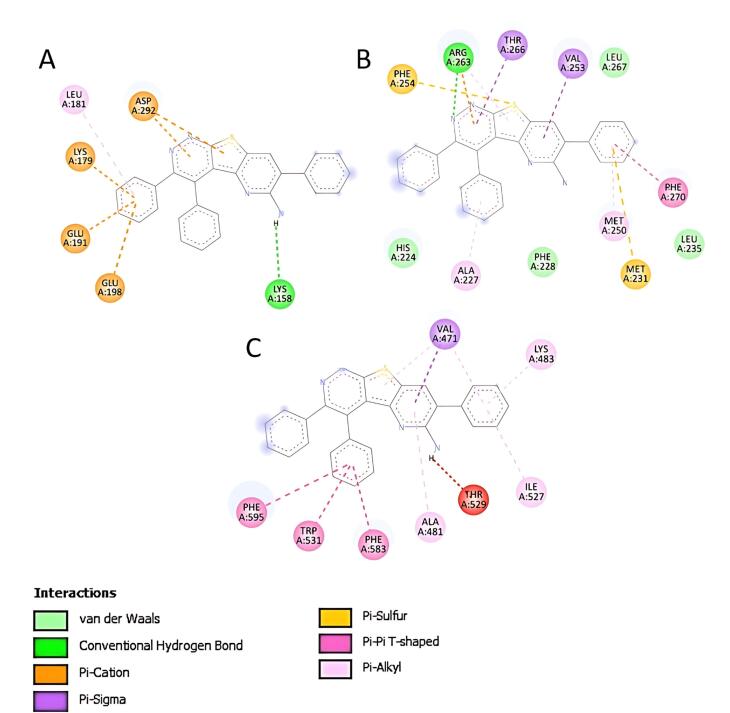
Binding interactions between compound **4 **and residual amino acids of AKT-1protein (4GV1) (A), MCL-1 protein (5FDO) (B) and BRAF protein (5VAM) (C)

 The high binding affinity of compound **4** against AKT-1 (4GV1) resulted from π and conventional hydrogen bond interactions with amino acid residues (Figure 3A). Compound **4 **also showed π, van der Waals and conventional hydrogen bonds against MCL-1 protein (5FDO) (Figure 3B). In addition, π binding was responsible for the interaction between compound **4 **and BRAF protein (5VAM) (Figure 3C). Overall these interactions and the molecular size of compound **4** followed Lipinski’s rule, thus providing reasonable values for pharmacokinetic parameters in drug development.^10^

## Conclusion

 The study successfully determined secondary metabolites of *P. caroliniana* and *P. millegrana*to have significant cytotoxicity contributions based on OPLS-DA analysis. Computational approaches suggested compound **4** of *P. caroliniana* as a biomarker compound responsible for the cytotoxicity against HeLa-cell protein marker. This straightforward protocol can be applied in biomarker discovery in other medicinal plants without excessive phytochemical experiments.

## Competing Interests

 None.

## Ethical Approval

 Not applicable.
